# A Study in Heads

**Published:** 1895-08

**Authors:** Julius Pohlman

**Affiliations:** Buffalo, N. Y., Professor of physiology, Medical Department, University of Buffalo.


### A STUDY IN HEADS.

By JULIUS POHLMAN, M. D., Buffalo, N. Y.,
Professor of physiology, Medical Department, University of Buffalo.

THERE was a time not very long ago when phrenology was fashionable and accepted by a fair majority of mankind as a certain method to determine any latent ability or capacity in men, women and children. There was a time when parents took their offspring to the "Professor" to be told that the boy had in him the making of a Webster, a Grant or a Lincoln; or that the girl would shine as a great leader of fashion, of thought, of society. Fathers, and more so mothers, were willing to pay high fees to have their vanity tickled, for who would not willingly sacrifice something to know that his or her boy or girl would outshine anybody else's boy or girl? The absurdity of the thing which presupposed that the "Professor," a perfect stranger, could, by merely looking at the child, tell the parents something about that child's ability which they had not yet discovered themselves, did not appear until later.

As the scientific localisation of brain functions progressed, phrenology has been relegated to the curiosities of the past, but yet we run across stray remnants of it quite frequently, and probably the commonest form of these is the often-heard saying, "what a fine head!" Just as if the head in its totality had any more to do with the mind inhabiting that head, than the town-lot-like arranged map of the phrenologist had to do with the determination of brain functions.

The question, "what is a fine head?" is yet to be answered, and the following note may add a trifle to the interest involved in both question and answer.

As this is a Buffalo number to celebrate the jubilee of a Buffalo journal, this study has been based on heads of Buffalo citizens, successful men excepting one, No. 4, and more or less well known.

Messrs. Georger & Co., the well-known firm of hatters and furriers, have for years collected a large series of forms of heads incidental to their business, and from this collection the following groups have been selected as fair types of large numbers of others. The figures are all drawn with the forehead toward the upper edge of the page and their right and left sides on the right and left side of the reader.

*Group I.*—These are fair types of dolichocephalic heads. Nos. 1 and 2, Germans; No. 3, an American—merchant, grocer and merchant respectively. No. 4 represents the head of a young German normal school student who constantly suffered from nervous troubles.

*Group II.*—This group represents heads with a more or less pointed occipital region—heads in which the posterior one-third is smaller in area than either one of the other thirds. What a lovely type of prize-fighter, according to phrenology, No. 5 would be with the temporal regions so excessively developed, but Mr. W. is a peace-loving young German of a meek and amiable disposition; No. 6 is a German merchant; No. 7 an American lawyer, and No. 8 an American lawyer and judge of high and well-deserved fame.

*Group III.*—These four specimens can well be called types of round heads—all Germans. Nos. 9 and 12 merchants; No. 10 an organist and composer, and No. 11 a physician.

*Group IV.*—Nobody will care to contradict the statement that Nos. 13–16, in group IV., represent very irregularly shaped heads. No. 13 is a real estate dealer of Irish parentage; No. 14 a German liquor dealer; No. 15 a German teacher of high standing, and No. 16 a German mechanic, a man of artistic tastes, a fine shot and sportsman.

It is very tempting to speculate upon the "whys" of these different forms of heads. The only thing certain, however, is that fifteen out of these sixteen represent good citizens and men successful in their respective occupations. Nevertheless, it cannot be doubted that if the skulls from which the above drawings have been made had been exhumed from some pre-historic burial-ground, the anthropologists would be greatly puzzled to determine the racial affinities and the mental caliber of the men whose remnants they were examining.

382 Franklin Street.

